# Perforation of a Retrocecal Appendix Resulting in Retroperitoneal Air: A Case Report

**DOI:** 10.1155/2013/584925

**Published:** 2013-02-11

**Authors:** Behrang Litkouhi, Alicia S. Huang, David J. Lundy, Maria Solis

**Affiliations:** Departments of Radiology and Surgery, Cooper University Hospital, Cooper Medical School of Rowan University, One Cooper Plaza, B23, Camden, NJ 08103, USA

## Abstract

There have been several case reports documenting acute appendicitis complicated by perforation presenting with retroperitoneal abscess formation. To date, there are no case reports of acute appendicitis in which the only sign for retroperitoneal perforation is the presence of retroperitoneal air as detected by computed tomography (CT). In the case presented, an 18-year-old male presented to the emergency department with clinical symptoms of acute appendicitis. CT exam demonstrated an inflamed appendix with multiple collections of air in the retroperitoneum, without abscess. Laparotomy revealed perforation of a retrocecal appendix into the retroperitoneum.

## 1. Introduction

Acute appendix, when complicated by perforation, typically presents with intraperitoneal, extraluminal air or abscess formation, which can be readily detected by CT. Several case reports have been published discussing cases of acute appendicitis with perforation resulting in retroperitoneal abscess. In all of these published accounts the appendix has had a retrocecal location. With perforation, the appendix presumably perforates through the posterior parietal lining of the peritoneum. To date, there are no case reports documenting acute appendicitis in which the only sign for retroperitoneal perforation is the presence of retroperitoneal air as detected by CT. The case presented here documents such a case.

## 2. Case Report

The patient is an 18-year-old male with no significant past medical history. He presented to the emergency department with a one-day history of suprapubic and periumbilical pain and nausea, without vomiting. On physical examination, he demonstrated mild periumbilical, right lower quadrant, and left upper quadrant tenderness to palpation. He was afebrile, with respiratory rate, heart rate, and blood pressure all within normal limits. A complete blood count revealed leukocytosis (20.6 × 10^9^/L) with a left shift. A diagnosis of acute appendicitis was considered, and CT exam was ordered. 

**Figure 1:**
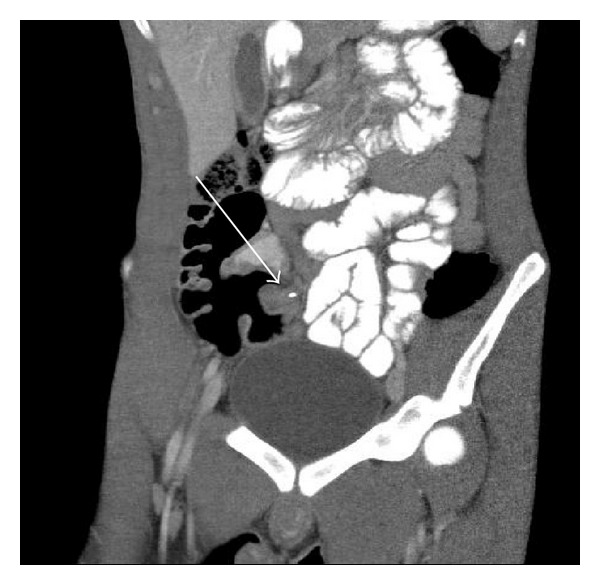
Coronal CT of the abdomen demonstrates dilated, thick-walled appendix (arrow) containing an appendicolith.

**Figure 2:**
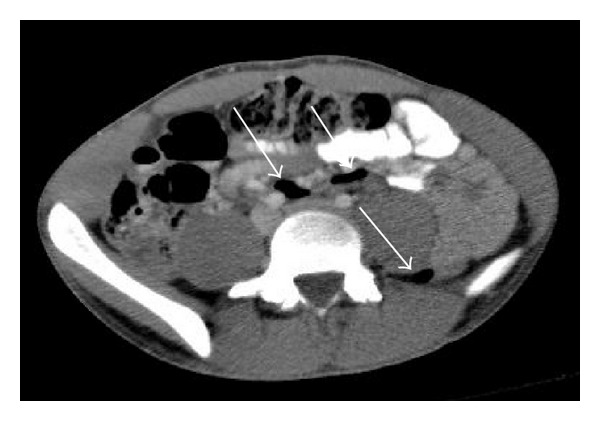
Axial CT demonstrates retroperitoneal air (arrows) anterior to the right and left common iliac arteries, and posterior to the left psoas muscle.

**Figure 3:**
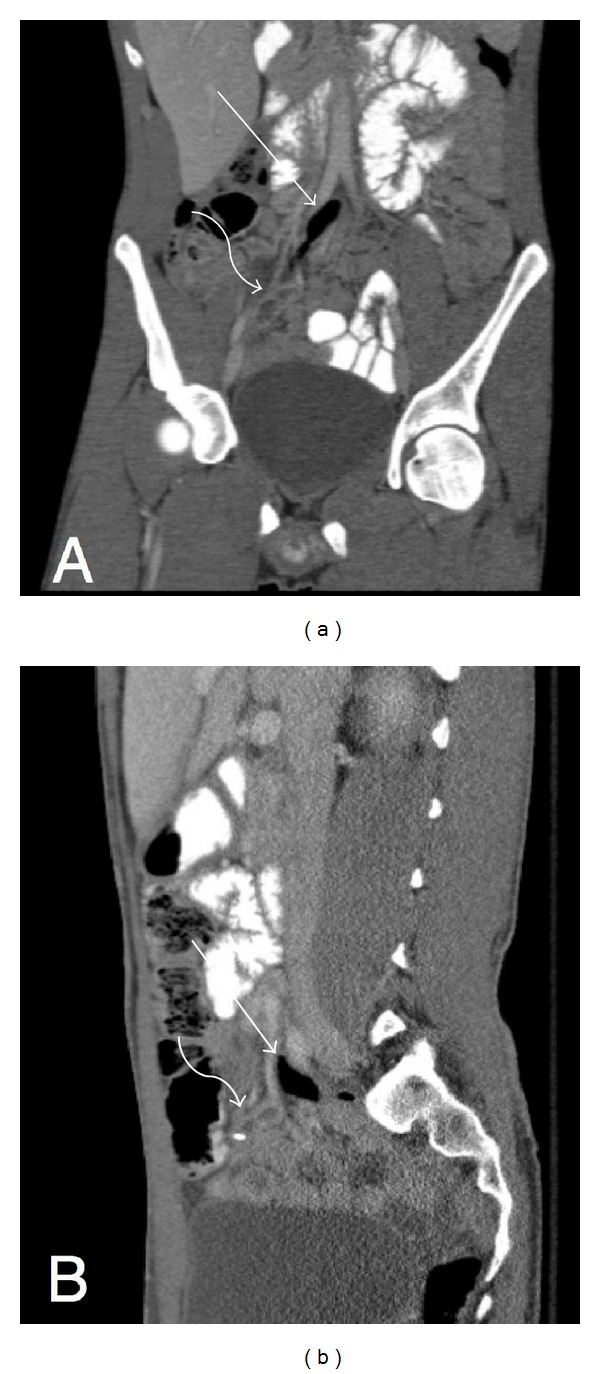
Retroperitoneal air (straight arrow) adjacent to inflamed, retrocecal appendix (curved arrow), seen on coronal (a) and sagittal (b) projections.

CT exam demonstrated a dilated, hyperenhancing, retrocecal appendix containing an appendicolith, indicative of acute appendicitis (Figure 1). In addition, small collections of air were seen anterior to the right and left common iliac arteries and posterior to both psoas muscles, locations that are considered retroperitoneal (Figures 2 and 3). There was no evidence of retroperitoneal abscess. No intraperitoneal free air was identified. The diagnosis of appendicitis with retroperitoneal perforation was considered; however, other causes for retroperitoneal air were not excluded. The patient was taken to the operating room for appendectomy and exploratory laparotomy. A lower midline surgical incision was performed instead of a right lower quadrant incision, to better evaluate for a possible concomitant retroperitoneal process. During surgery, an inflamed, retrocecal appendix was identified, with evidence of microperforation into the retroperitoneum. No additional retroperitoneal pathologic process was identified. Appendectomy was performed and the patient was discharged the next day in stable condition.

## 3. Discussion

There are several published accounts of acute appendicitis complicated by retroperitoneal abscess. A summary of all case reports published between 1955 and 2007 identified in a PubMed search was published by Hsieh et al. in 2007. Among the 24 documented cases, there was a mortality rate of 16.7%. Of the patients who did survive, all hospital stays were greater than 2 weeks [1]. Other case reports have documented acute appendicitis complicated by retroperitoneal perforation with abscess extending to the thigh, perinephric space, and the mediastinum [2–5]. Considering the extensive complications and relatively high mortality rate associated with retroperitoneal perforation of acute appendicitis, it is important to be able to recognize the early signs. In the case presented here, the only sign apparent on CT exam was retroperitoneal air. We postulate that, in this case, CT exam was performed early in the course of perforation, and therefore only retroperitoneal air was seen, without abscess.

Another important observation gathered from the several published accounts of patients with acute appendicitis with retroperitoneal abscess is that these patients often present with atypical and less severe abdominal complaints. This can delay diagnosis, which may contribute to the higher rates of complications and mortality. We propose that, with increasing use of CT exam early in the course of clinical presentation, more cases of acute appendicitis with perforation into the retroperitoneum presenting with retroperitoneal air may occur, leading to earlier interventions and more successful clinical outcomes.
